# Exoskeleton Training and Trans-Spinal Stimulation for Physical Activity Enhancement After Spinal Cord Injury (EXTra-SCI): An Exploratory Study

**DOI:** 10.3389/fresc.2021.789422

**Published:** 2022-01-04

**Authors:** Tommy W. Sutor, Mina P. Ghatas, Lance L. Goetz, Timothy D. Lavis, Ashraf S. Gorgey

**Affiliations:** 1 Spinal Cord Injury and Disorders Center, Hunter Holmes McGuire Veteran Affairs Medical Center, Richmond, VA, United States,; 2 Department of Physical Medicine and Rehabilitation, Virginia Commonwealth University, Richmond, VA, United States

**Keywords:** exoskeleton, trans-spinal stimulation, body composition, oxygen uptake, EMG, spinal cord injury, rehabilitation

## Abstract

After spinal cord injury (SCI) physical activity levels decrease drastically, leading to numerous secondary health complications. Exoskeleton-assisted walking (EAW) may be one way to improve physical activity for adults with SCI and potentially alleviate secondary health complications. The effects of EAW may be limited, however, since exoskeletons induce passive movement for users who cannot volitionally contribute to walking. Trans-spinal stimulation (TSS) has shown the potential to enable those with even the most severe SCI to actively contribute to movements during EAW. To explore the effects of EAW training on improving secondary health complications in persons with SCI, participants with chronic (*n* = 8) were enrolled in an EAW program 2–3 times per week for 12 weeks. Anthropometrics (seated and supine waist and abdominal circumferences (WC and AC), body composition assessment (dual exposure x-ray absorptiometry-derived body fat percent, lean mass and total mass for the total body, legs, and trunk), and peak oxygen consumption (VO_2_ during a 6-minute walk test [6MWT]) were assessed before and after 12 weeks of EAW training. A subset of participants (*n* = 3) completed EAW training with concurrent TSS, and neuromuscular activity of locomotor muscles was assessed during a 10-m walk test (10MWT) with and without TSS following 12 weeks of EAW training. Upon completion of 12 weeks of training, reductions from baseline (BL) were found in seated WC (−2.2%, *P* = 0.036), seated AC (−2.9%, *P* = 0.05), and supine AC (−3.9%, *P* = 0.017). Percent fat was also reduced from BL for the total body (−1.4%, *P* = 0.018), leg (−1.3%, *P* = 0.018), and trunk (−2%, *P* = 0.036) regions. No effects were found for peak VO_2_. The addition of TSS for three individuals yielded individualized responses but generally increased knee extensor activity during EAW. Two of three participants who received TSS were also able to initiate more steps without additional assistance from the exoskeleton during a 10MWT. In summary, 12 weeks of EAW training significantly attenuated markers of obesity relevant to cardiometabolic health in eight men with chronic SCI. Changes in VO_2_ and neuromuscular activity with vs. without TSS were highly individualized and yielded no overall group effects.

## INTRODUCTION

Spinal cord injury (SCI) results in physical impairment and limited mobility. For people with the most severe SCI, wheelchairs are necessary for mobility, leaving persons with SCI vulnerable to subsequent chronic comorbidities (1, 2). These comorbidities may include psychosomatic, cardiovascular, metabolic consequences, and a significant socioeconomic burden (1, 3–6). The economic burdens for persons with SCI, their families, and society are increasing at an alarming rate, especially with decreasing mortality due to advances in medical interventions (7). Restoring mobility may serve as a potential rehabilitation intervention that cuts down several of the SCI-associated comorbidities and associated economic burdens (8).

Historically, several rehabilitation modalities to restore and improve locomotion following SCI have been used, such as long-leg braces, hip-knee-ankle foot orthosis, Parastep powered by functional electrical stimulation, body weight-supported treadmill training, and robotic treadmills. In fact, several of these techniques were considered interventions to counterbalance changes in body composition, reduced level of physical activity, and abnormal lipid and carbohydrate profiles (8–14). Unfortunately, most of these rehabilitation interventions require too high of metabolic energy demand for individuals with SCI to tolerate. Thus, despite the potential success at meeting various goals of some of these interventions, lack of widespread adoption leaves many persons with SCI unlikely to meet recommended physical activity guidelines to overcome inactivity-induced health deficits (15).

Another potential tool to help persons with SCI meet physical activity guidelines is powered robotic exoskeletons, the use of which has greatly expanded in recent years (16–20). Exoskeletons offer a unique opportunity for persons with SCI to experience standing and walking at a low metabolic cost (16, 21–23). Exoskeleton-assisted walking (EAW) may decrease spasticity, improve bowel movements, and improve physical activity levels (14, 24). A systemic review concluded that exoskeletons provide a safe, easy to use, and a practical method of neurorehabilitation in persons with SCI (16). Another review highlighted several of the applications that are likely to benefit and enhance the quality of life in persons with SCI (25). Previous studies reported that a frequency of 2–3 times per week or more for 1–2 h may be beneficial in the rehabilitation of persons with SCI (21, 26).

The impact of exoskeletons on the cardiovascular profile is still equivocal, unlike modalities, such as neuromuscular electrical stimulation, which likely enhance one’s overall cardiovascular profile after SCI. One reason is that exoskeletons move the legs of the users passively if they are not able to volitionally contribute to locomotion, as is the case for persons with severe SCI—thus, muscle contractions may be minimal or non-existent, so the magnitude of the cardiovascular effect is unclear. Noninvasive trans-spinal stimulation (TSS) is one strategy that has consistently shown enhancement of neuromuscular activity below the level of SCI (27), such as enhancing active stepping during EAW (28, 29). If TSS+EAW can enhance neural and by extension muscular activity, then theoretically it should increase metabolic activity and therefore has enhanced positive effects on body composition—however, these outcomes have not been tested.

As an initial step toward conducting larger clinical trials, the purpose of this pilot study was to explore the effects of EAW on body composition, oxygen consumption, and physical activity in persons with chronic SCI. Outcome measures included anthropometric circumferences, whole-body and regional body composition assessments using dual-energy x-ray absorptiometry (DXA), peak oxygen consumption, and neuromuscular activity during EAW. In a subset of participants, the acute effects of TSS on peak muscle activation and the ability to step with reduced exoskeleton assistance were explored. Lastly, for participants who received TSS, the number of steps that could be completed without vs. with full assistance from the exoskeleton during TSS vs. no-TSS conditions was assessed.

## METHODS

The current study was approved by the Institutional Review Board at the Hunter Holmes McGuire VA Medical Center. The study was registered on clinicaltrials.gov (EXTra-SCI; NCT03410550).

### Participants

Individuals were eligible for inclusion if they were between 18 and 70 years of age, at least 1-year post-SCI, and were medically cleared by the study doctor to participate, which included meeting manufacturer specifications to participate in EAW. Participants (*n* = 8 men) were enrolled after giving informed consent. Details of the participant are presented in Table 1.

### Outcome Measures

Anthropometric, body composition, and oxygen consumption outcomes were assessed at baseline (BL) and post-intervention (PI) after 12 weeks of EAW. Tests to assess the effects of training on electromyography (EMG) activity and the effects of TSS were tested at PI. The minimum exoskeleton-provided assistance level achieved by each participant over the course of training is also reported. Blood pressure and heart rate were monitored for safety throughout testing and all training sessions.

### Anthropometrics

Waist and abdominal circumference were measured in both seated and supine positions using a tape measure. Waist circumference was measured at the narrowest region of the trunk, while abdominal circumference was measured at the level of the umbilicus. For both assessments, measurements were taken twice, and if they differed by more than 1 cm, extra measurements were taken until a reliable measurement was obtained (13, 30, 31).

### Body Composition

Dual-energy x-ray absorptiometry scans were used to assess trunk, leg, and total body percent fat mass and lean mass using a Lunar Prodigy Advance (Lunar Inc., Madison, WI, USA). After calibration, scans were performed by a trained and certified DXA operator using Lunar software, version 10.5. After laying supine for 20 min, the legs were strapped proximal to the knees and ankles, with the arms and legs positioned to ensure proper alignment during the scan. Participants remained still for ~10 min during scanning. The coefficient of variability of two repeated scans is <3% (32).

### EAW Training Protocol

For this study, all participants used a powered exoskeleton, which can provide variable levels of assistance to the user (EksoGT or EksoNR, Ekso Bionics, CA, USA). Participants were engaged in EAW twice- or thrice-weekly for 12 weeks. These exoskeletons can provide an adjustable level of swing-phase assistance during walking. Participants initially received 100% of motor support that could be provided by the exoskeleton during the swing phase. Each week, participants were tested to see if they could progress to a lower level of assistance by walking 10 m with the exoskeleton set to deliver 5% less assistance than the week before. Audible tones provided by the exoskeleton during walking indicated whether a step was finished with or without extra motor output from the exoskeleton. If a participant completed two 10-m walking bouts with 80% or greater steps completed at the set assistance level without additional assistance, the participant completed their training sessions that week at the newly achieved assistance level.

### Walking: Clinical and Other Outcomes

A 6-minute walk test (6MWT) was used to test oxygen consumption (VO_2_) during EAW. After being fitted in the exoskeleton, participants sat resting for 5 min. A mask was then snugly fitted to their face to measure VO_2_ with a portable metabolic cart (COSMED K4b2, Concord, CA, USA). The peak VO_2_ was recorded for the following conditions and durations: sitting (5 min) and standing (5 min), 6MWT (6 min), standing recovery (3 min), and sitting recovery (3 min).

A 10-m walk test (10MWT) was used to test the effects of training and TSS on peak EMG activation during EAW. At PI, two tests were performed while walking with the exoskeleton providing 100% assistance, and two tests were performed while walking with the exoskeleton providing the minimal amount of assistance achieved through training (Min%). For participants who received TSS, these conditions were tested with and without the presence of TSS. The percentage of steps during the final 10MWT that required extra assistance from the exoskeleton with vs. without TSS was also quantified. Surface EMG activity of the right-leg vastus lateralis, semitendinosus, medial gastrocnemius, and soleus muscles was recorded during 10MWT testing using wireless Trigno Avanti Sensors (Delsys Incorporated, Natick, MA, USA). An electrogoniometer affixed to the right knee of the exoskeleton was used to record the knee angle. All signals were collected at 2,000 Hz and stored on a laptop computer for offline analysis in MATLAB (The MathWorks Inc., Natick, MA, USA). EMGs of both trials of each condition were visually inspected, and the trial for each condition, which had the clearest EMG bursts, was selected for analysis. The knee angle was used to visually segment strides (defined as the period from initiation of knee flexion to the next initiation), and strides were then further segmented into swing and stance phases. Raw EMGs were then band-passed (fourth-order Butterworth, 25–400 Hz). For participants who received TSS, the TSS artifact was removed by applying a comb filter, as done previously (33, 34). Signals were then smoothed using a root-mean-square envelope with a moving window of 100 samples. The peak amplitude for every stride and each swing and stance phase for each muscle was then obtained. The peak amplitude of the whole stride was considered a separate variable since the peak muscle activity did not consistently occur in only stance or swing phase across and within participants. Peak amplitudes for each step were then averaged to yield an average peak EMG stride, stance, and swing amplitude for each participant for each condition.

### TSS Protocol

Trans-spinal stimulation was delivered to a subset of participants with motor complete injuries (*n* = 3; participants 004, 005, and 006). Other participants with motor complete injuries (003, 0772, and 0773) were enrolled in the EAW-only portion of the study. To initially determine tolerance and responsiveness of each participant to TSS, participants lay supine with a single 10.2 × 17.8 cm cathode electrode placed posteriorly to cover the lumbosacral segment (L1–S2; the distal end of the electrode was placed at L4/L5 space and the proximal end reaches to T0/T11 space) centered over the vertebral column (ValuTrode, AxelGaard Manufacturing Co., Ltd, Fallbrook, CA, USA). Two 5.1 × 8.9 cm electrodes were placed bilaterally over the anterior iliac crests as anodes. Using monophasic 1 ms pulses at 30 Hz (DS7AH, Digitimer, Fort Lauderdale, FL, USA), the stimulation amplitude was gradually increased until evoked responses were visible on the lower extremity EMGs. The lowest amplitude at which muscle activity occurred in supine was used during EAW to facilitate the initiation of stepping. During EAW, a clinically available stimulator (TheraTouch EX4, Richmar, Clayton, MO, USA) was used to deliver TSS with the same electrode configuration that was used for mapping. Biphasic 2 ms pulses (1 ms phase duration, no interphase interval) were used to minimize charge build up under any one electrode during EAW sessions.

### Statistical Analysis

Anthropometric, body composition, and VO_2_ results are presented as means ± SD, along with percent change from BL. Two participants (003 and 007) were unable to complete PI testing due to the halting of research activities during the COVID-19 pandemic. BL data for these participants were included, and multiple imputation was used to include these participants in an intent-to-treat analysis when possible. Due to limited sample size and variability in the data, non-parametric-related sample Wilcoxon sign-rank tests were used to compare changes from BL to PI. Differences were considered significant at *P* ≤ 0.05. Peak EMGs were compared between three different conditions to address three different questions concerning the effects of different factors on EMG activity. First, to test the effects of reduced exoskeleton-provided assistance, peak EMG amplitudes during EAW at Min% were normalized to amplitudes during EAW at 100% assistance. Second, to test the effects of TSS during EAW at 100% assistance, peak EMGs with TSS were normalized to peak EMGs without TSS. Third, to test the effects of TSS during EAW at Min%, peak EMGs with TSS were normalized to EMGs without TSS. Lastly, the percentage of steps during the final 10MWT that required extra assistance from the exoskeleton with vs. without TSS was quantified. Due to low participant numbers and the exploratory nature of EMG and extra-assistance step outcomes in this study, individual percent differences are presented addressing each question and discussed qualitatively without inferential statistics.

## RESULTS

### Anthropometrics

**FIGURE 1 |:**
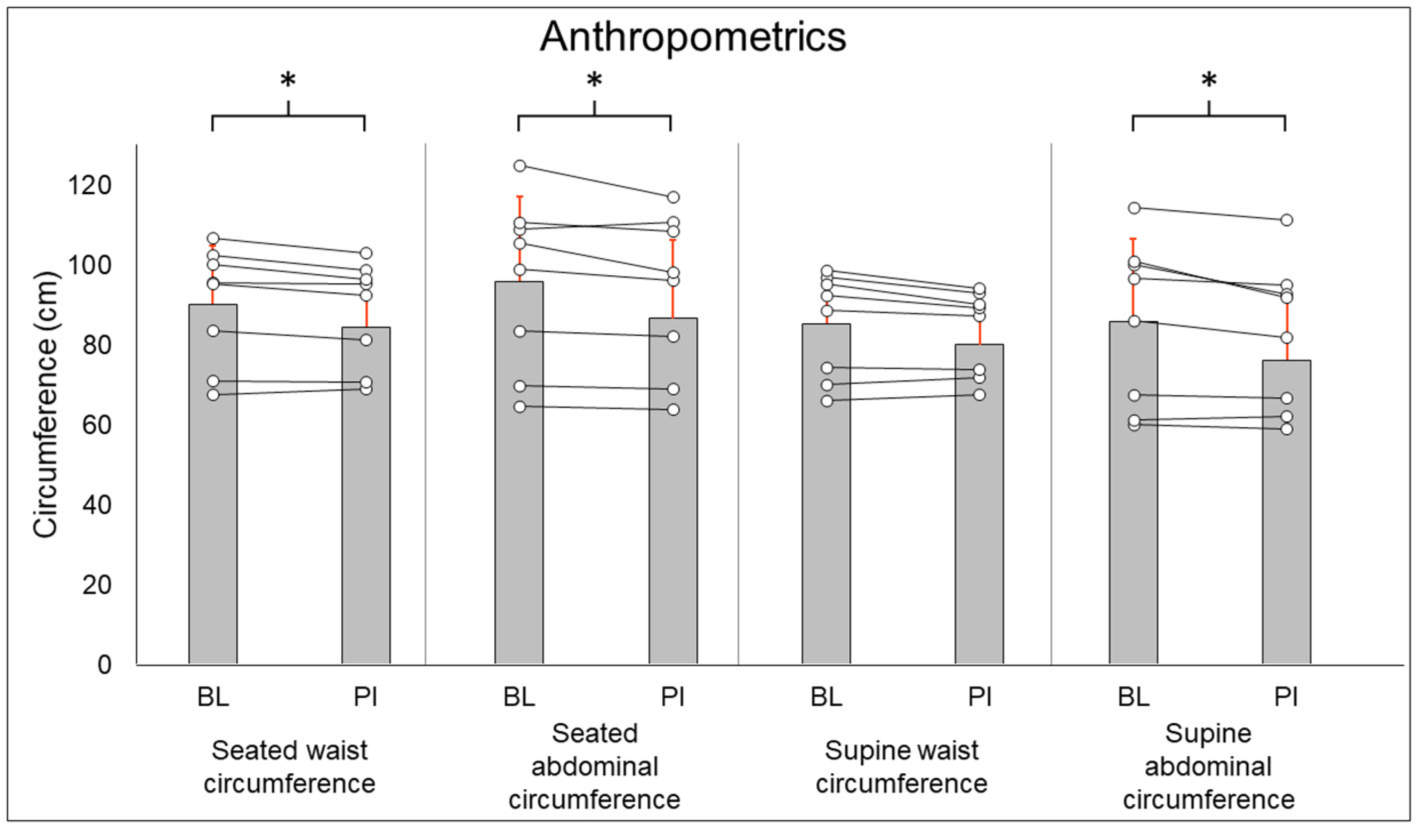
Anthropometric results. Gray bars represent group averages (red lines showing SD), with dots connected by lines representing individual participants and their changes from baseline to post-intervention measurements. BL, baseline; PI, post-intervention. *Significant to *P* < 0.05.

Anthropometric results are summarized in Figure 1. Seated waist circumference (BL: 90.2 ± 14.6 cm, PI: 88.3 ± 13.0 cm [−2.1%], *P* = 0.036), seated abdominal circumference (BL: 95.9 ± 21.2 cm, PI: 93.1 ± 19.6 cm [−2.9%], *P* = 0.05), and supine abdominal circumference (BL: 85.9 ± 20.5 cm, PI: 82.5 ± 18.5 cm [−3.9%], *P* = 0.02) all were decreased after training. Supine waist circumference (BL: 85.3 ± 13.0 cm, PI: 83.3 ± 10.5 cm [−2.2%], *P* = 0.123) did not change after training.

### Body Composition

**FIGURE 2 |:**
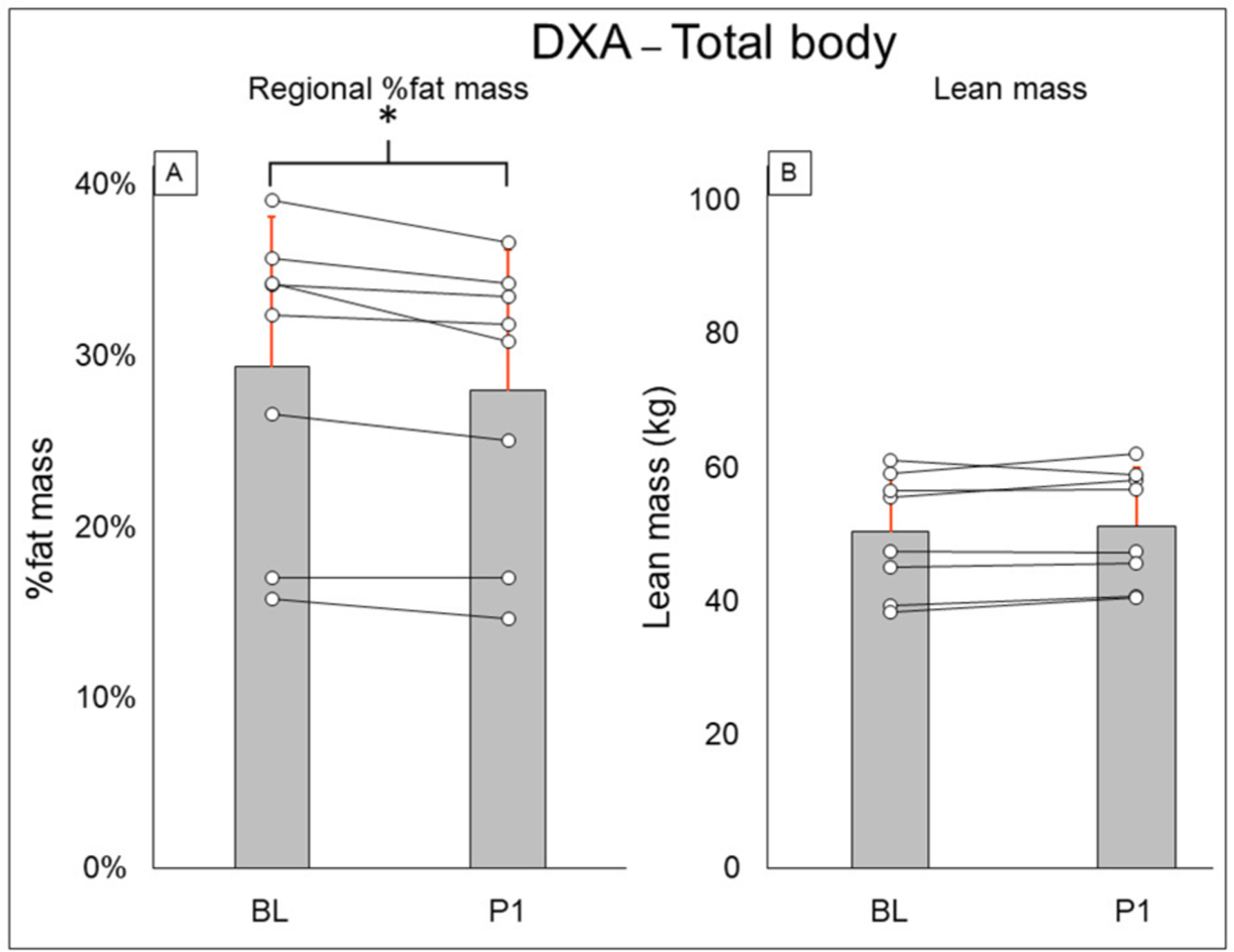
Total body DXA results for regional % fat mass **(A)** and lean mass **(B)**. Gray bars represent group averages (red lines showing SD), with dots connected by lines representing individual participants and their changes from baseline to post-intervention measurements. DXA, dual-energy x-ray absorptiometry; BL, baseline; PI, post-intervention. *Significant to *P* < 0.05.

**FIGURE 3 |:**
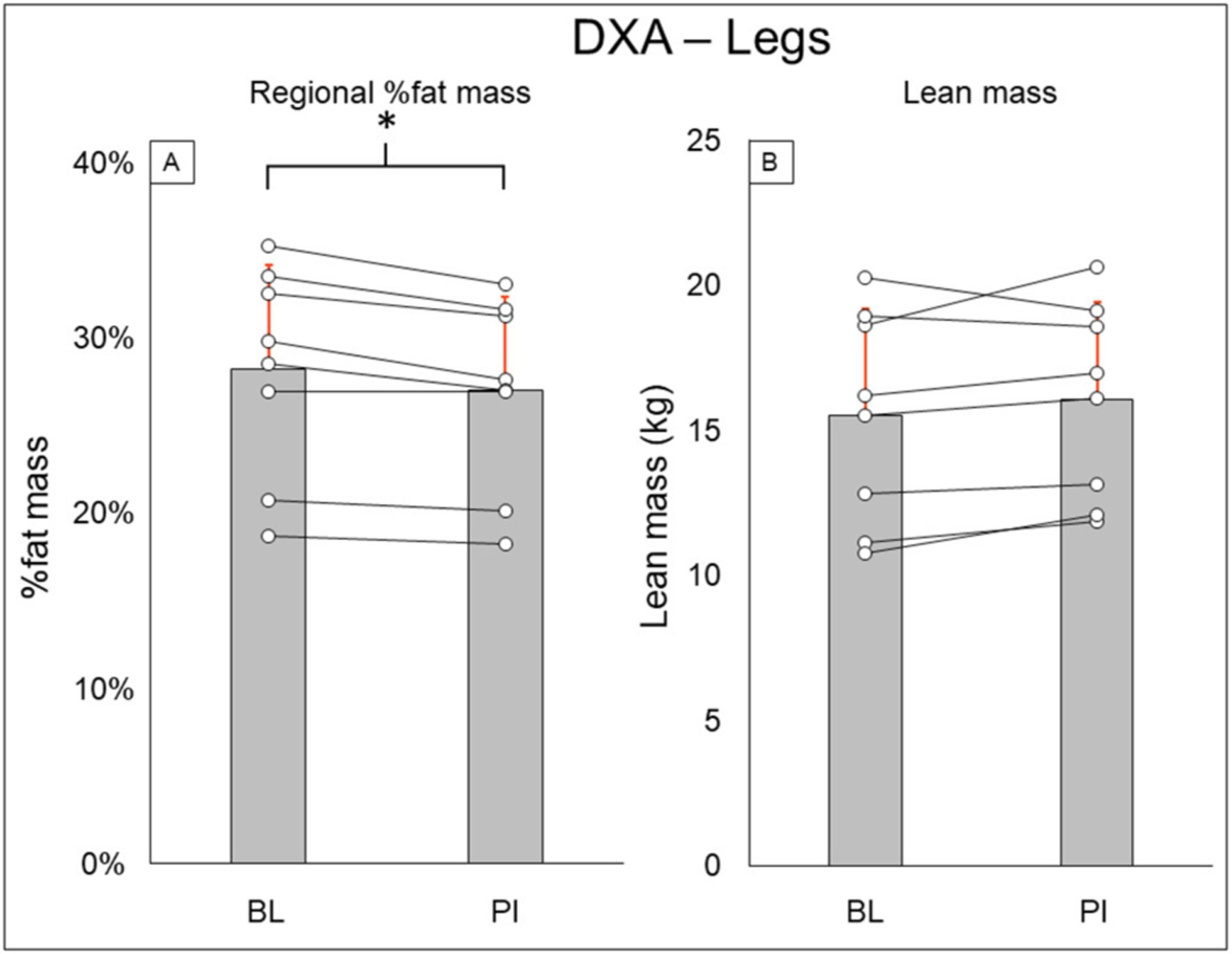
Leg DXA results for regional % fat mass **(A)** and lean mass **(B)**. Gray bars represent group averages (red lines showing SD), with dots connected by lines representing individual participants and their changes from baseline to post-intervention measurements. DXA, dual-energy x-ray absorptiometry; BL, baseline; PI, post-intervention. *Significant to *P* < 0.05.

**FIGURE 4 |:**
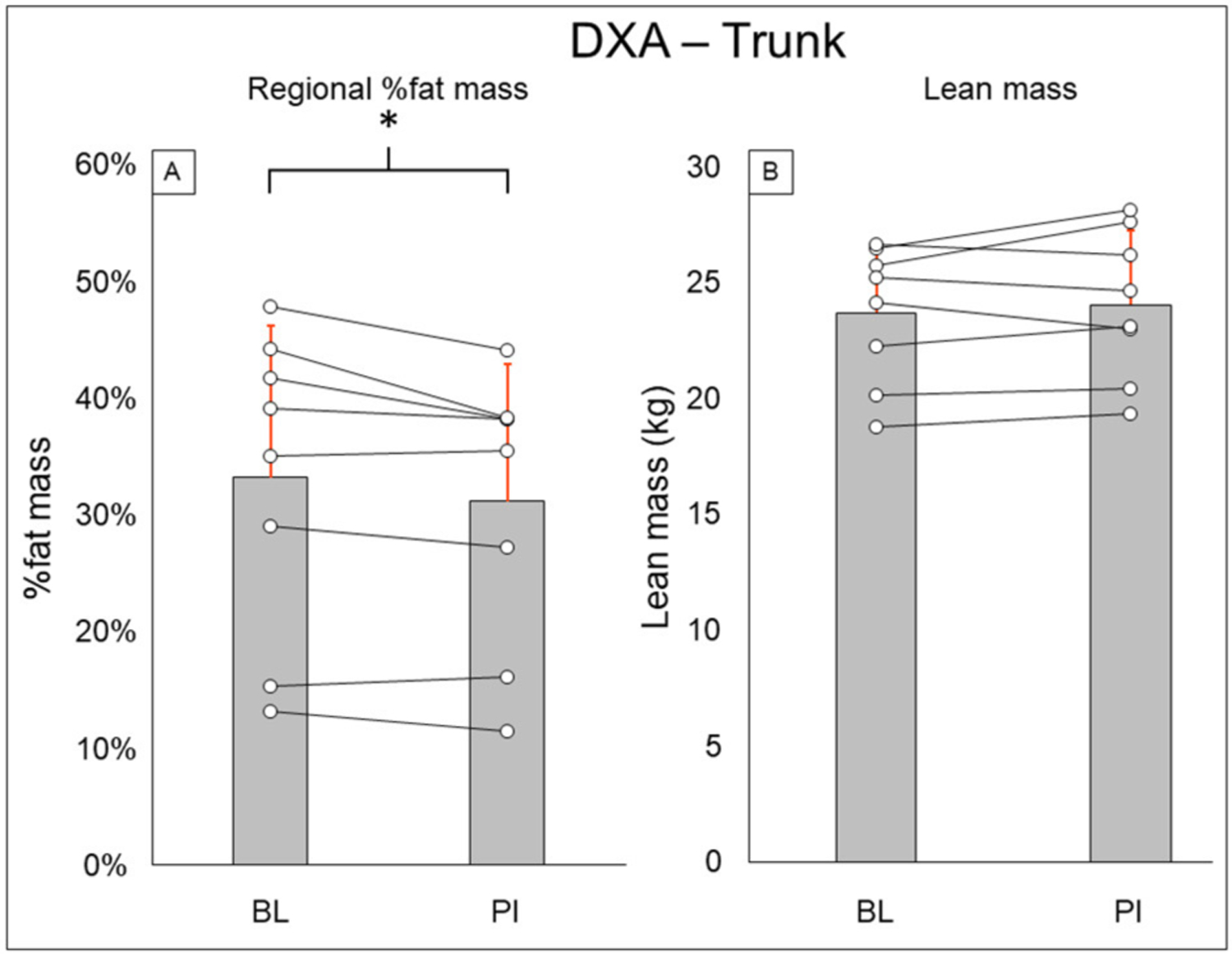
Trunk DXA results for regional % fat mass **(A)** and lean mass **(B)**. Gray bars represent group averages (red lines showing SD), with dots connected by lines representing individual participants and their changes from baseline to post-intervention measurements. DXA, dual-energy x-ray absorptiometry; BL, baseline; PI, post-intervention. *Significant to *P* < 0.05.

Total-body DXA results are summarized in Figure 2. Total body percent fat mass (BL: 29.3 ± 8.7%, PI: 27.9 ± 8.2% [−1.4%], *P* = 0.02) was significantly decreased after training. Total body lean mass (BL: 50.3 ± 8.9 kg, PI: 51.2 ± 8.7 [+1.9%], *P* = 0.123) and total body mass (BL: 77.5 ± 20.5 kg, PI: 75.8 ± 18.1 [−2.1%], *P* = 0.263) did not change after training. Leg DXA results are summarized in Figure 3. Leg percent fat mass (BL: 28.2 ± 5.9%, PI: 26.9 ± 5.3% [−1.3%], *P* = 0.018) was significantly decreased after training. Leg lean mass (BL: 15.5 ± 3.6 kg, PI: 16.1 ± 3.4 kg [+3.4%], *P* = 0.16) and leg total mass (BL: 23.2 ± 6.1 kg, PI: 23.4 ± 5.9 kg [+1%], *P* = 0.4) did not change after training. Trunk DXA results are summarized in Figure 4. Trunk percent fat mass (BL: 33.1 ± 13%, PI: 31.1 ± 11.7% [−2%], *P* = 0.036) was significantly decreased after training. Trunk lean mass (BL: 23.7 ± 3.0 kg, PI: 24 ± 3.2 kg [+1.5%], *P* = 0.4) and trunk total mass (BL: 38.8 ± 11 kg, PI: 37.7 ± 9.8 kg [−2.8%], *P* = 0.4) did not change after training.

### Oxygen Consumption

**FIGURE 5 |:**
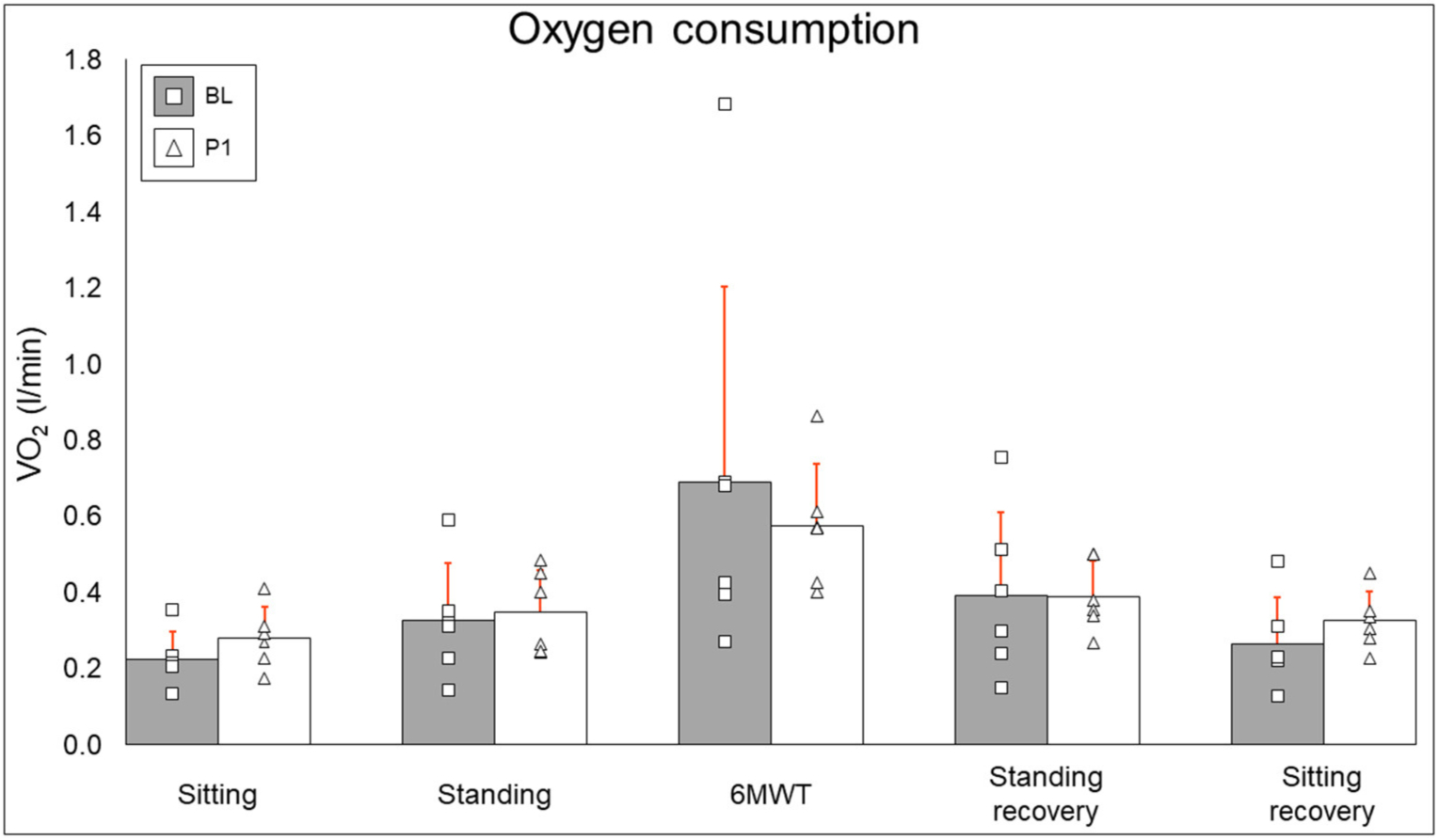
Oxygen consumption results. Bars represent group averages (gray for baseline, white for post-intervention–red lines showing SD) with squares and triangles representing individual baseline and post-intervention values, respectively. l/min, liters per minute; BL, baseline; PI, post-intervention.

Oxygen consumption results are summarized in Figure 5. Resting (BL: 0.226 ± 0.072 l/min, PI: 0.281 ± 0.08 l/min [+24.3%], *P* = 0.12), standing (BL: 0.327 ± 0.151 l/min, PI: 0.349 ± 0.11 l/min [+6.6%], *P* = 0.46), 6MWT (BL: 0.691 ± 0.513 l/min, PI: 0.574 ± 0.166 l/min [−16.9%], *P* = 0.6), standing recovery (BL: 0.393 ± 0.218, PI: 0.391 ± 0.093 [−0.7%], *P* = 0.92), and sitting recovery (BL: 0.266 ± 0.121 l/min, PI: 0.325 ± 0.76 l/min [+22.2%], *P* = 0.12) all did not change after training.

### Exoskeleton-Provided Assistance and EMG Activity—Effects of Training

The minimum percent of exoskeleton-provided swing-phase assistance achieved through training is presented for each participant in Table 1. Statistical analysis was not performed for this outcome as it is presented for informational purposes only (see section Discussion for further explanation).

**FIGURE 6 |:**
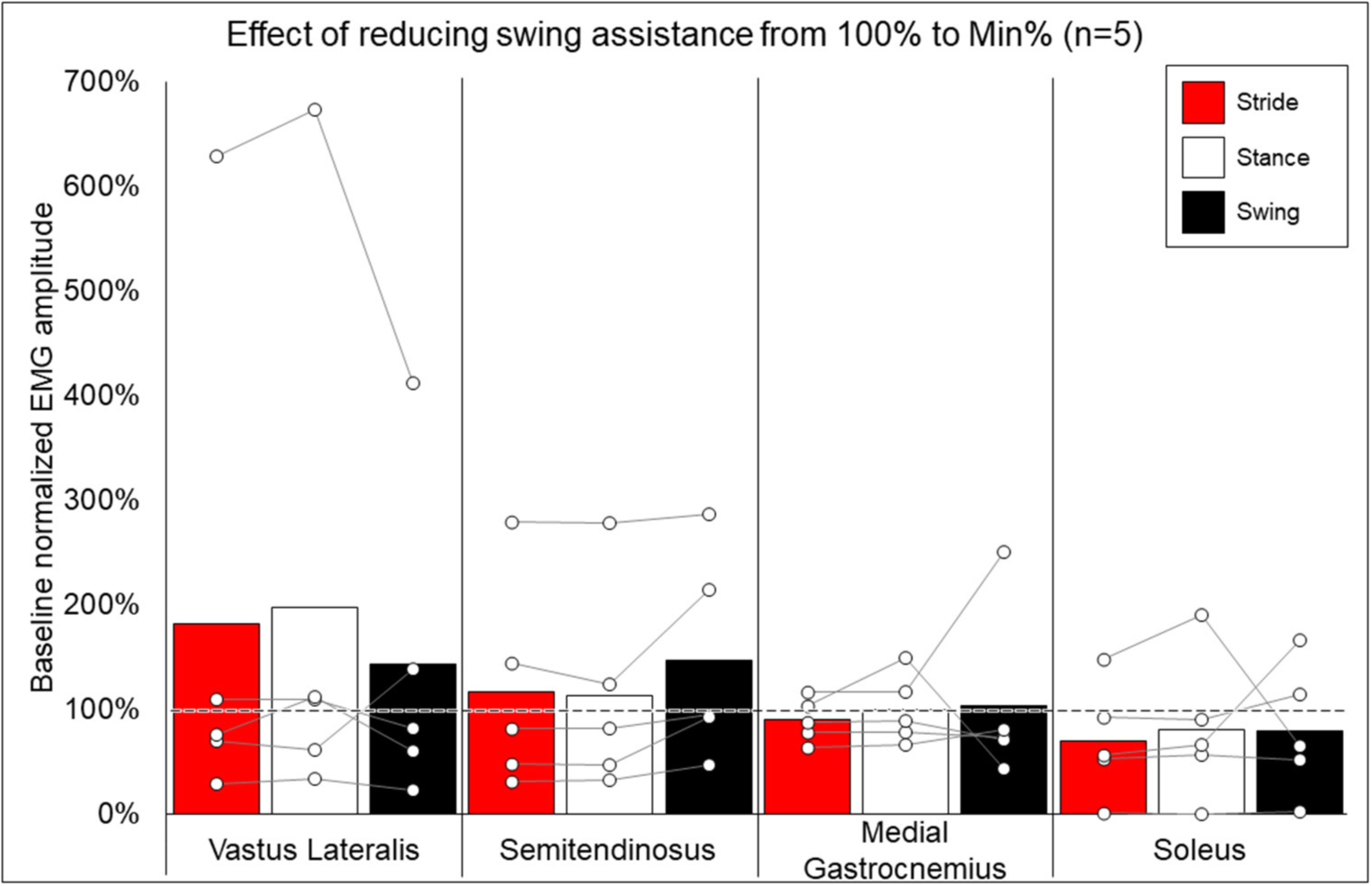
Effects of reducing swing assistance from 100% to the minimum percent (Min%) achieved through training on select locomotor muscles. Bars representing group averages and dots representing individual participants show EMG amplitudes during Min% normalized as a percent of average EMG amplitudes when walking at 100% assistance (represented by the dotted line on the graph).

Methods for EMG collection were expanded to be more comprehensive after the first participant, and post-training EMG data are not available for two participants, thus data from five participants were used to compare the change in peak EMG activity when walking at minimal assistance from walking at 100% assistance. Figure 6 shows the average and individual peak EMG activity during EAW at the minimum assistance attained during training normalized to the EMG activity during EAW with 100% assistance from the exoskeleton.

### EMG Activity—Effect of Addition of TSS

**FIGURE 7 |:**
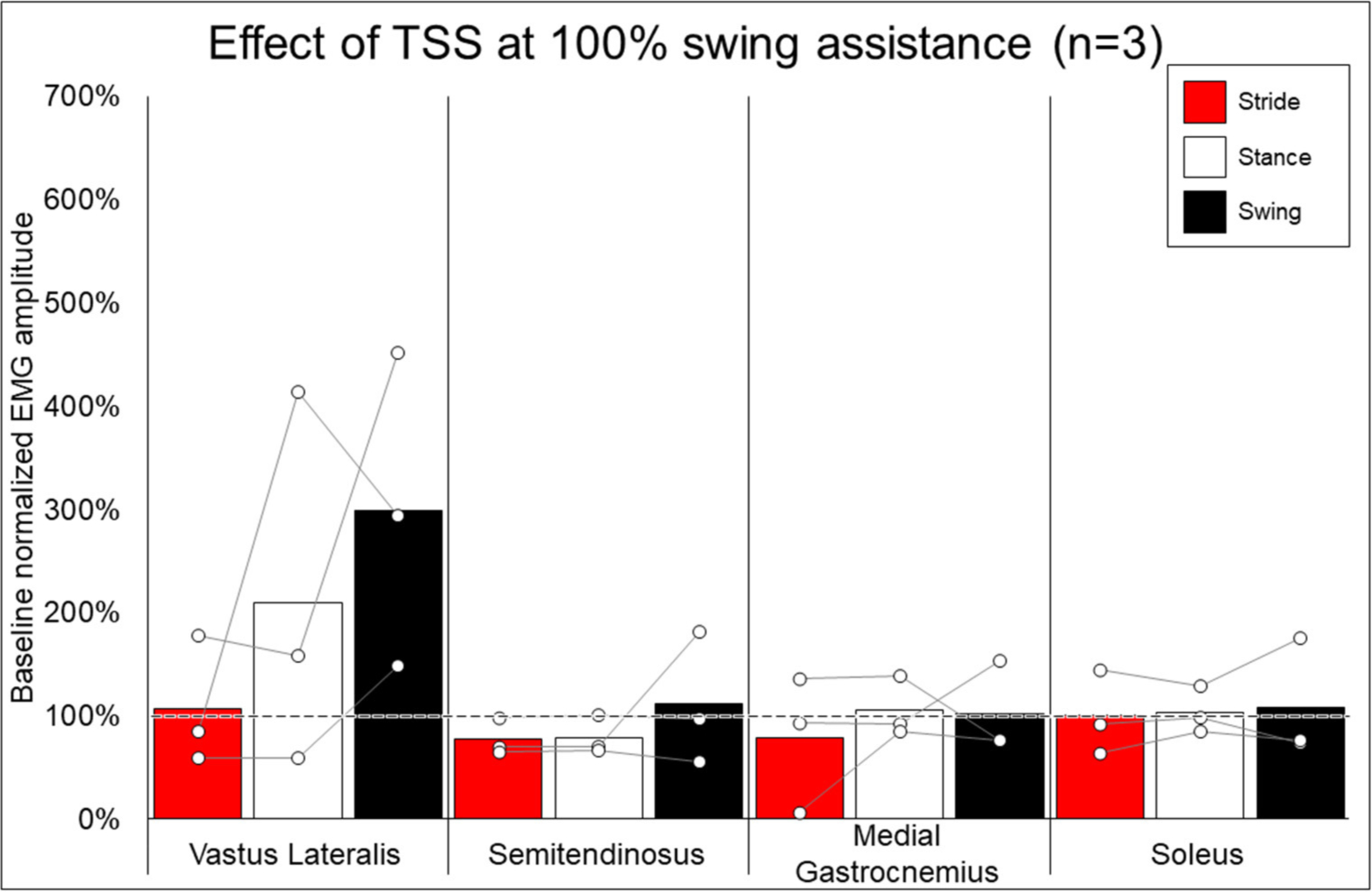
Effect of adding trans-spinal stimulation to exoskeletal assisted walking at 100% swing assistance. Bars representing group averages and dots representing individual participants show EMG amplitudes during Min% normalized as a percent of average EMG amplitudes when walking at 100% assistance (represented by the dotted line on the graph).

**FIGURE 8 |:**
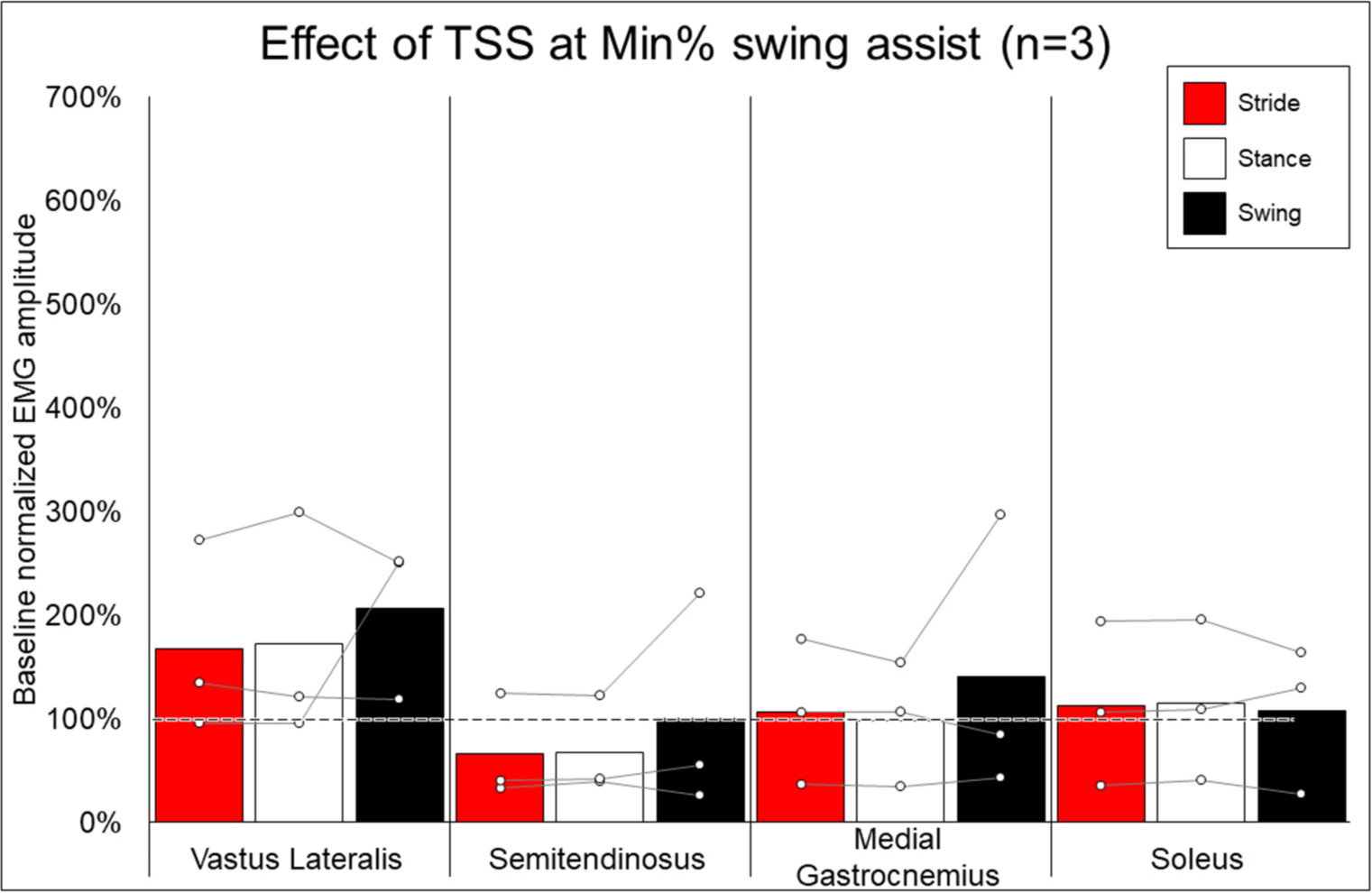
Effect of adding trans-spinal stimulation to exoskeletal assisted walking at minimum percent assistance achieved through training. Bars representing group averages and dots representing individual participants show EMG amplitudes during Min% normalized as a percent of average EMG amplitudes when walking at 100% assistance (represented by the dotted line on the graph).

**FIGURE 9 |:**
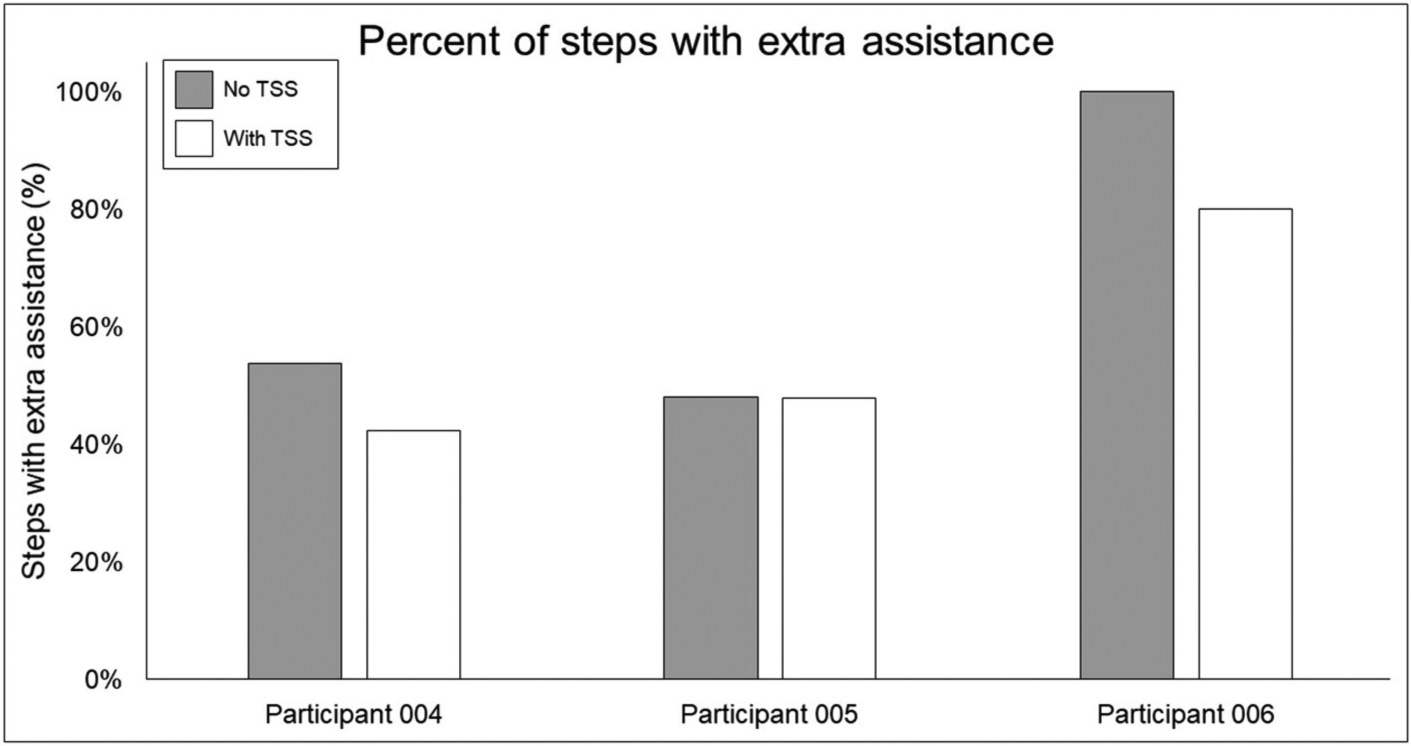
Percentage of steps requiring extra swing assistance from the exoskeleton beyond the minimum percentage achieved through training. Gray bars show the percentage of steps during a 10MWT without (gray bars) vs. with (white bars) trans-spinal stimulation—a lower value indicates that fewer steps in a given bout required extra assistance beyond the programmed level in the exoskeleton. TSS, trans-spinal stimulation.

Figure 7 shows the average and individual peak EMG activity during EAW at 100% assistance with TSS normalized to the EMG activity during EAW at 100% assistance without TSS. Figure 8 shows the average peak EMG activity during EAW at the minimum assistance achieved through training with TSS normalized to the EMG activity during EAW at minimum assistance without TSS. Figure 9 shows the percentage of steps during the same 10MWT in which the EMGs were collected that the exoskeleton provided audible tones to indicate that extra assistance was provided to the user to complete a step.

## DISCUSSION

This study was carried out to provide preliminary data necessary to calculate effect sizes and determine power to design future EAW and TSS clinical trials. The sample size was chosen to explore the effect of EAW on anthropometrics, body composition, and VO_2_–attempts to recruit a larger sample were halted with the onset of the COVID-19 pandemic. The addition of TSS on this small scale was an initial step to examine the hypothesis that we can shift the paradigm of passive EAW (i.e., complete reliance on exoskeleton-provided assistance) into a more active-assisted paradigm to allow participants to actively contribute to EAW (28, 29). Twelve weeks of twice- or thrice-weekly EAW yielded significantly improved seated waist and abdominal circumference, supine abdominal circumference, and total body percent fat mass.

### Anthropometrics and Body Composition

Supine and seated waist and abdominal circumferences are associated with increased risk of cardiometabolic issues in persons with SCI, such as excessive visceral adipose tissue, dyslipidemia, and inflammation (35–37). Total body fat percentage is also related to cardiometabolic risk factors, such as dyslipidemia, hypertension, and dysfunctional glucose metabolism (38). Previously, once-weekly EAW training was found to not have an impact on body composition, despite increasing energy expenditure and improving physical activity (20). Given that participants in this study doubled or tripled the weekly training frequency compared to this previous study, favorable changes in these anthropometric and body composition measures may be expected. Importantly, the reductions seen in regional % fat for the total body, legs, and trunk (−4.7, −4.5, and −6.2%, respectively) are all greater than estimated long-term precision errors for DXA scans (32). Thus, these significant reductions in fat and significant reductions in three of four anthropometric circumferences support that participants in this EAW training program had meaningful reductions in central adiposity that could improve overall cardiometabolic health.

Along with reductions in fat mass, the lack of an effect on lean mass may also be expected. An increase in lean mass has been detected with loading and with muscle stimulation during locomotor training. The EAW program in this study focused on working toward taking as many steps as possible with the lowest assistance from the exoskeleton that was achievable. Thus, the EAW training program did not involve loading that would lead to muscle hypertrophy or increase lean mass in other ways.

### Oxygen Consumption

Twelve weeks of EAW did not change resting, walking, or recovery oxygen consumption. It is critical to note that while participants put forth a maximal effort to cover as much distance as possible during the 6MWT, a 6MWT during EAW has been shown to elicit only sub-maximal VO_2_ values in people with motor complete SCI (21). Thus, a difference in VO_2_ outcomes from BL to PI may not occur. Additionally, inter-participant differences in this study, such as the minimum level of exoskeleton-provided assistance achieved during walking or engaging in EAW with or without TSS, will likely influence the variability of VO_2_ response to EAW across 12 weeks of training. With these factors notwithstanding, it is noteworthy that even though our EAW training paradigm focused on minimizing assistance rather than maximizing speed, participants in this study still experienced an increase in VO_2_ from resting during EAW, potentially producing an aerobic exercise effect sufficient to bring about the favorable changes seen in anthropometrics and body composition.

### EMG Activity and the Effects of TSS

While group averages of EMG data are presented to give a comprehensive summary of the data, inter-participant variability is quite apparent. Often, group averages are clearly influenced by participants who, relative to other participants, had an exceptionally large increase (006, quadricep and hamstring activation) or decrease (0773, soleus activity) when walking at 100% vs. minimum assistance, as seen in Figure 6. Fundamentally, these data show that varying the level of exoskeleton-provided assistance will yield highly individual responses. These individual responses may depend on multiple factors, such as training status or walking kinematics delivered by the exoskeleton at different levels of assistance (39). Importantly, the level of exoskeleton-provided assistance to enable users to walk may reflect volitional effort from the user yet is also influenced by the weight of the legs of the users (heavier limbs require more torque from the exoskeleton) or spasticity (strong flexion or extension spasms may impede the exoskeleton freely moving the legs in a walking pattern). This explains not only why many participants with motor-complete SCI showed reduced exoskeleton-provided assistance through training (seen in Table 1) but these may also be additional factors influencing individual EMG results. Extensive investigation of these factors was beyond the scope of this paper and further investigation is necessary.

In looking at the effects of TSS on either 100% or minimal exoskeleton assistance, some general patterns emerged from the data despite the low number of participants who received TSS. Among the variable responses seen, it is noteworthy that in both exoskeleton assistance conditions, all participants experienced increased EMG activity in the quadriceps during the swing phase of gait with TSS (Figures 8, 9). In most cases, hamstring activity decreased or remained unchanged, with the one exception being participant 004 while walking with minimal assistance from the exoskeleton. These results largely agree with previous a previous report showing that TSS mainly modulates the activity of proximal locomotor muscles in an individual with motor complete SCI during EAW (28). For the distal gastrocnemius and soleus muscles, considerable inter-participant variability is apparent. As covered in the last paragraph, a comprehensive investigation of factors affecting this variability was beyond the scope of this pilot study, though the data seem to suggest that the most reliable effect of TSS is modulation of proximal muscle activity.

In terms of the functional significance of altered muscle activity, when walking with minimal assistance from the exoskeleton, two out of three participants achieved a greater amount of steps without extra exoskeleton assistance (above the fixed minimal assistance level) with TSS vs. without TSS. When considering peak EMG activity, the only similarity between these two participants (004 and 006) is they both showed increased peak EMG activity during the whole stride and stance and swing phases (these participants were dissimilar for age, weight, injury level, time since injury, and AIS classification; see Table 1), whereas participant 005 only showed an increase during the swing phase. It is possible that increasing peak muscle activity of a crucial weight-bearing muscle is what enabled these participants to take more steps without extra exoskeleton assistance—further investigation is necessary to clarify this finding.

## LIMITATIONS

Conclusions that can be drawn from this study are limited by the small sample size, and some outcomes are limited to sub-groups of participants. The use of multiple imputation in limited sample size is a valid technique to fill in missing data (40) and similar to what our group has published previously (41). The authors recognize, however, that even with multiple imputation, data in this study were limited and the significant findings for anthropometrics and body composition must be confirmed in larger sample sizes. The dietary habits and physical activity outside of the study were also not controlled, which could have affected the body composition results. However, all participants enrolled in the trial were complete SCI with more than 1-year post-injury. Specific to our TSS outcomes, the small sample size of participants who received TSS allowed exploration to refine TSS protocols. Thus, conclusions cannot be drawn from these results, though this trial allowed our research group to explore stimulation techniques to enhance EMG activity during EAW. This information also served as preliminary data to help to plan a currently approved clinical trial (NCT04241250). Our TSS technique also did not use a carrier frequency, which may offer an added benefit over simpler square-wave waveforms (42).

## CONCLUSION

This study showed that 12 weeks of EAW can improve anthropometrics and body composition in persons who are non-ambulatory due to chronic SCI. These effects may partially be due to the aerobic exercise enabled by EAW, though EAW itself seems to cause variable aerobic adaptations between individuals. Altering the level of exoskeleton-provided assistance may impact muscle activation, and the addition of TSS may further modulate muscle activation, potentially in a way that enables the user to contribute more actively to EAW.

## Figures and Tables

**TABLE 1 |:** Participant characteristics enrolled in the current trial.

Participant ID	Age	Weight (kg)	Height (cm)	LOI	BMI	TSI (yrs)	AIS	Min%
001	26	50	184	C6	14.8	2	C	100%
003	54	97	166	T4	35.1	13	B	100%
004	22	75	187	C5	21.5	7	B	60%
005	32	65	183	C7	19.4	8	A	55%
006	52	92	184	T4	27.1	21	A	55%
007	54	98	177	T10	31.2	5	C	80%
0772	25	49	174	C8	16.0	5	A	55%
0773	36	99	182	T11	29.9	7	B	70%
Mean	37.6	78.1	179.8	NA	24.4	8.5	NA	
SD	13.7	21.5	6.8	NA	7.5	6.0	NA	

LOI, level of injury; BMI, body mass index; TSI, time since injury; AIS, American spinal injury association Impairment Scale; Min%, minimum percent of exoskeleton-provided swing-phase assistance achieved through training.

## Data Availability

The raw data supporting the conclusions of this article will be made available by the authors, without undue reservation.
